# Prevalence and clinical characterisation of thyroid dysfunction in COPD: a systematic review and meta-analysis

**DOI:** 10.3389/fmed.2025.1571165

**Published:** 2025-04-30

**Authors:** Ling Wu, Li He, Xiaorong Hu, Hanqiong Zhang, Zeming He, Xiaotao Huang, Caihong Li, Yong Zhang

**Affiliations:** ^1^Department of Respiratory and Critical Care Medicine, The First People’s Hospital of Shuangliu District, Chengdu, Sichuan, China; ^2^Department of Thoracic Surgery, The First People’s Hospital of Shuangliu District, Chengdu, Sichuan, China; ^3^Department of Traditional Chinese Medicine, The First People’s Hospital of Shuangliu District, Chengdu, Sichuan, China; ^4^Department of Nursing, The First People’s Hospital of Shuangliu District, Chengdu, Sichuan, China

**Keywords:** chronic obstructive pulmonary disease, thyroid function, meta-analysis, systematic review, non-thyroidal illness syndrome (NTIS)

## Abstract

**Background:**

The prevalence of COPD is increasing annually, accompanied by a growing number of complications and organ function abnormalities. Thyroid dysfunction is prevalent among patients with chronic obstructive pulmonary disease (COPD). Updated evidence is needed to complement previous systematic reviews on this topic to provide best practice.

**Methods:**

The EMBASE, Web of Science, Cochrane and PubMed databases were searched for articles containing the keywords “COPD” and “thyroid dysfunction” (PROSPERO CRD42024592606). Eligibility screening, data extraction and quality assessment of retrieved articles were performed independently by two reviewers. Meta-analyses were performed to determine the prevalence of thyroid dysfunction in patients with COPD. Regression analyses were used to explore sources of heterogeneity. The clinical features of COPD combined with thyroid dysfunction were clarified by comparing the age, sex (percentage of males), BMI, smoking index, Forced Vital Capacity (FVC%), Forced Expiratory Volume in One Second (FEV1%), partial pressure of oxygen (PaO2), partial pressure of carbon dioxide (PaCO2), and albumin in patients with and without thyroid dysfunction. The differences in the prevalence of thyroid dysfunction between stable and acute exacerbations in COPD were also compared.

**Results:**

Twelve studies were included, with an overall prevalence of 42.1% (95% CI, 31.8–52.9). The most common type of thyroid dysfunction in COPD was non-thyroidal illness syndrome (NTIS) in 45.3% (95% CI, 22.3–68.3). There was no difference in the prevalence of dysfunctions between stable and acute exacerbations of COPD. Patients in the thyroid dysfunction group in COPD had lower PCO2 and albumin and higher FEV1%.

**Conclusion:**

Thyroid dysfunction has a high prevalence among patients with COPD, with NTIS being the most common. Thyroid dysfunction in COPD may affect lung function and lead to decreased albumin. Patients with COPD should be screened for thyroid function, and attention should be paid to the clinical features of this group of patients with thyroid dysfunction to facilitate better identification and management.

**Systematic review registration:**

https://www.crd.york.ac.uk/PROSPERO/myprospero, PROSPERO ID (CRD42024592606).

## 1 Introduction

Chronic Obstructive Pulmonary Disease (COPD) is a heterogeneous lung disease characterised by chronic respiratory symptoms due to persistent (usually progressive) airflow obstruction due to airway abnormalities (bronchiectasis, bronchiectasis minor) and/or alveoli (emphysema), which are manifested mainly by dyspnea, cough, and sputum (1). The pulmonary function index Forced Expiratory Volume in One Second as a percentage of predicted value (FEV1%) is used to assess the severity of airflow limitation. The main etiological factors are inhalation of harmful particles and gases, such as smoking and haze (1). COPD has a high prevalence in the population and has became the third leading cause of death globally in 2020, significantly increasing healthcare burden. Nowadays, more and more studies have shown that due to long-term hypoxia, carbon dioxide retention, systemic inflammation and other factors in patients with chronic obstructive pulmonary disease (COPD), their lesions are not only limited to the lungs, but also involve multiple systems and organs of the whole body, with a variety of comorbidities or complications, such as cardiovascular disease (coronary heart disease), lung cancer, diabetes mellitus, metabolic syndrome, disorders of endocrinological system, anaemia, osteoporosis, and psychological disorders (anxiety), and so on (2). Among them, thyroid disorders are common in patients with COPD (3). Uzun et al. concluded that severe airway obstruction and excessive respiratory muscle loading affect thyroid hormone levels in patients with COPD (4). Tobacco and smoke, which are also considered major risk factors for COPD, may also cause thyroid hormone abnormalities (5). Some studies have reported that serum TSH levels are negatively correlated with length of hospital stay (6), and are even a determinant of the frequency of acute exacerbations of COPD (7). Plasma fT3 levels are considered to be a reliable indicator of the severity of disease in patients with acute respiratory failure in COPD (8), and low serum fT3 and fT4 concentrations increase the morbidity and mortality of patients with respiratory failure on invasive mechanical ventilation, suggesting that thyroid hormone levels may influence the prognosis of AECOPD (9). These findings suggest that thyroid function should be closely monitored in COPD patients, which is beneficial for assessing the condition and prognosis of COPD patients and better managing such patients to promote their rapid recovery. Currently, there are fewer data on the prevalence of thyroid dysfunction in COPD, only one systematic evaluation summarized the prevalence of thyroid dysfunction in COPD (10), which included just nine studies and did not perform a heterogeneity analysis. On the basis, this paper builds upon that foundation by adding relevant literature and data, thereby expanding the sample size. It not only investigates the prevalence of thyroid dysfunction among COPD patients but also examines the prevalence of the most common type, Non-Thyroidal Illness Syndrome (NTIS). Furthermore, it compares the prevalence of thyroid dysfunction during stable COPD and acute exacerbation phases and analyzes the clinical characteristics of patients with thyroid dysfunction in COPD. Additionally, this paper explores the possible mechanisms underlying the occurrence of thyroid dysfunction in COPD and its impact on prognosis, which is beneficial for clinicians in identifying critically ill COPD patients and assessing their prognoses.

## 2 Methodology

### 2.1 Search strategy

The Preferred Reporting Items for Systematic Evaluation and Meta-Analysis (PRISMA) guidelines were consulted for this systematic evaluation and meta-analysis, and the protocol for this systematic evaluation was registered with PROSPERO (CRD42024592606). The search strategy included a combination of Medical Subject Headings (MeSH) terms and keywords related to thyroid dysfunction and COPD. Four databases (EMBASE, PubMed, Web of Science, Cochrane) were searched from their inception to 16 August 2024. Relevant reviews and references of included studies were manually searched for other possible studies (see Appendix 1).

### 2.2 Study selection

Articles were included if they reported on the prevalence of thyroid dysfunction in patients with COPD. Types of articles included observational studies with cross-sectional, case–control, and cohort designs. Articles published as narrative reviews, conference abstracts, and case reports were excluded. Both COPD and thyroid dysfunction must be diagnosed using valid and objective methods. For example, spirometry for COPD. Two reviewers (Ling Wu and Li He) independently screened the titles and abstracts of all relevant articles for eligibility, and consensus was reached through discussion when disagreements were encountered. Then, the two reviewers mentioned above reviewed the full text, extracted and analysed the data, and compared and analysed the results, and when disagreements that could not be resolved through discussion were encountered, a third reviewer (Xiaorong Hu) was consulted. When necessary, we also contacted the authors of some publications to obtain more information about the methods and data.

### 2.3 Data extraction

Two reviewers (Ling Wu and Li He) independently used Microsoft Excel to create standardised spreadsheets to extract the appropriate required data from the included studies, including authors’ names, year of publication, country, type of study design, sample size, number of prevalent thyroid dysfunctions, demographic characteristics of the participants (age (in years), sex ratio (% of males), Body mass index (BMI (kg/m^2^), smoking index (pack/year)), clinically relevant indicators (Forced Expiratory Volume in One Second (FEV1) (%), Forced Vital Capacity (FVC) (%)), partial pressure of oxygen (PaO2) (mmHg), partial pressure of carbon dioxide (PaCO2) (mmHg) and albumin (g/L)). A table was created to compare the prevalence of Thyroid dysfunction between stable and acute exacerbations of COPD. They were resolved through discussion or consultation with the third reviewer (Hu Xiaorong). When disagreements arose, the two reviewers rechecked the conflicting data to identify the reasons for the disagreement. They determined the results through comparison and discussion. If the results remained inconsistent after the discussion, they consulted a third reviewer (Hu Xiaorong) to resolve the issue.

### 2.4 Quality assessment

Two reviewers (Ling Wu and Li He) independently assessed the quality of the final included articles (Quality Assessment Forms—Celiac Disease—NCBI Bookshelf). For the quality assessment of cross-sectional studies, the standards recommended by the Agency for Healthcare Research and Quality (AHRQ) were used (Appendix 2). This standard includes 11 items, with each item rated as “yes” “no” or “unclear.” A “yes” rating earns 1 point, while “no” or “unclear” ratings do not earn any points. The quality assessment criteria for the article are as follows: low quality = 0–3 points; moderate quality = 4–7 points; high quality = 8–11 points. For case–control studies and cohort studies, the Newcastle-Ottawa Scale (NOS) was utilized. The NOS scale has specific evaluation criteria designed for both types of studies (Appendices 3, 4). The NOS tool focuses on three areas: selection of study participants, comparability between groups, and outcome/exposure factors. In the selection of study participants, there are 4 items, with a maximum score of 4 points; for comparability between groups, there is 1 item, with a maximum score of 2 points; and for outcome/exposure factors, there are 3 items, with a maximum score of 3 points. The overall potential risk of bias has a maximum score of 9 points, with higher scores indicating a lower risk of bias. The quality assessment criteria are as follows: low quality = 0–3 points, moderate quality = 4–6 points, high quality = 7–9 points. When the two reviewers had inconsistent quality scores, they each re-evaluated the article’s quality scores and then discussed the discrepancies with each other. If they could not reach an agreement, they consulted a third reviewer (Hu Xiaorong) to resolve the issue.

### 2.5 Statistical analysis

Statistical analysis was performed using STATA software (version 18, STATA Inc., College Station, TX, United States). The prevalence of thyroid dysfunction in COPD patients was expressed as a percentage (%) for each included study. The Meta-prop method was enhanced by the Freeman-Tukey double arcsine transformation for variance stabilization, used to calculate the prevalence of thyroid dysfunction in the targeted studies. A random-effects model was employed to calculate the 95% confidence intervals (CIs) for the prevalence rates. Heterogeneity among the study results was determined using the chi-squared test and the I-squared statistic; significant heterogeneity was indicated by an I-squared value greater than 50% and a *P* value less than 0.05. If heterogeneity was present, meta-regression was applied to explore the sources of heterogeneity. Sensitivity analysis was conducted on the included studies to identify any studies that had a significant impact on the results. Publication bias was assessed using funnel plots and Egger’s test. If publication bias was observed, the trim-and-fill method was employed to adjust for publication bias.

## 3 Results

### 3.1 Study selection

**Figure 1:**
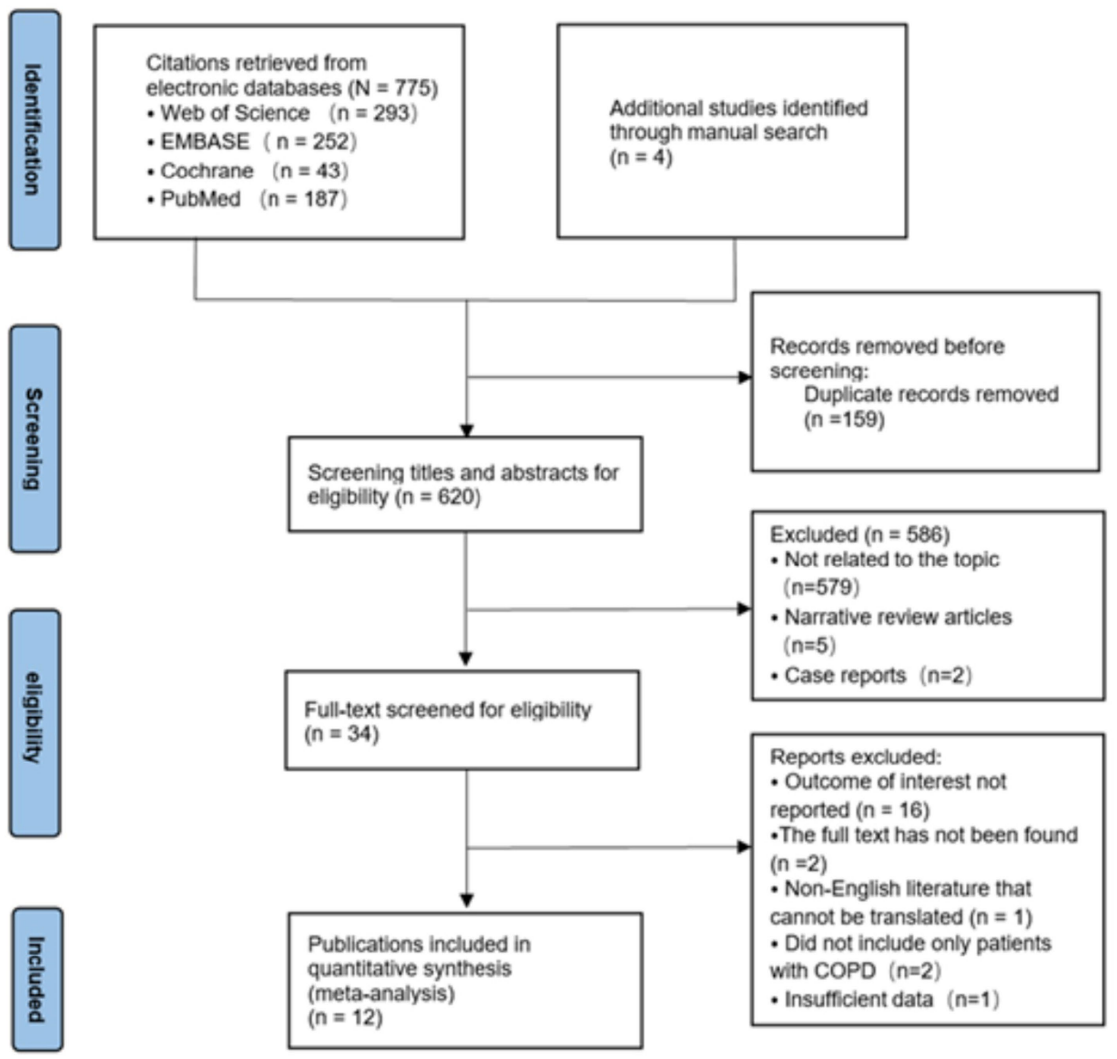
PRISMA flowchart of study selection.

Figure 1 shows the PRISMA study selection flowchart. A total of 775 articles were generated by searching the above databases through the search strategy. Four literature studies were added by manual search. After excluding 159 duplicates, titles and abstracts of 620 articles were screened for eligibility. Of these, the full text of 34 articles was screened for eligibility and a detailed assessment based on predetermined inclusion and exclusion criteria resulted in the identification of 12 studies (4, 6–8, 11–18) (Table 1). Seven of these studies were from Turkey, two from India, one from Iran, one from China, and one from Italy. Seven of the 12 studies were cross-sectional, one case–control, and four cohort studies. Table 2 shows the risk of bias assessment of the included studies. The assessment showed that there were no low-quality studies and the overall quality of the included studies was “high” and “medium” quality.

**Table 1:** Characteristics of included studies.

Study (first author/year)	Region	Study design	Sample size	Thyroid dysfunction (*n*)	Male (*n*(%))*	Age (Mean±SD) (y)
Uzun K 2007 (4)	Turkey	Cohort	62	20		64.5 ± 8.3
Karadag F 2007 (11)	Turkey	Cohort	103	43	103 (100)	66.52 ± 7.59
Yasar Z 2015 (12)	Turkey	Cohort	125	64	101 (80.8)	65 ± 11
Huang D 2021 (6)	China	Cohort	134	36	99 (73.88)	70.13 ± 10.03
Akbaş T 2024 (8)	Turkey	Cross-sectional	80	51	46 (57.5)	71.5 ± 9.5
Sarinc Ulasli S 2013 (7)	Turkey	Case control study	88	44	44 (50)	65.3 ± 6.65
Agin K 2013 (13)	Iran	Cross-sectional	34	8	34 (100)	51.74 ± 5.76
Bahçecioğlu S 2023 (14)	Turkey	Cross-sectional	78	53	70 (89.7)	68.60 ± 10.43
Gumus A 2021 (15)	Turkey	Cross-sectional	309	68	297 (96)	65.9 ± 9.8
Chaudhary SC 2018 (16)	India	Cross-sectional	171	43	126 (73.7)	57.75 ± 9.81
Verma S 2019 (17)	India	Cross-sectional	121	45	90 (74.38)	60.75 ± 8.89
Terzano C 2014 (18)	Italy	Cross-sectional	155	105	92 (59.35)	71.92 ± 10.97

* *n* represents the number of male patients in the study and % indicates the number of male patients as a percentage of the total.

**Table 2:** Quality assessment of included studies.

Study (first author/year)	Region	Study design	Selection	Comparability	Outcome	NOS score
Uzun K 2007 (4)	Turkey	Cohort	3	2	3	8
Karadag F 2007 (11)	Turkey	Cohort	3	2	3	8
Yasar Z 2015 (12)	Turkey	Cohort	4	2	3	9
Huang D 2021 (6)	China	Cohort	4	2	3	9
Akbaş T 2024 (8)	Turkey	Cross-sectional	3	2	3	8
Sarinc Ulasli S 2013 (7)	Turkey	Case control study	4	2	3	9

Study (first author/year)	Region	Study design	AHRQ score
Agin K 2013 (13)	Iran	Cross-sectional	7
Bahçecioğlu S 2023 (14)	Turkey	Cross-sectional	8
Gumus A 2021 (15)	Turkey	Cross-sectional	7
Chaudhary SC 2018 (16)	India	Cross-sectional	7
Verma S 2019 (17)	India	Cross-sectional	7
Terzano C 2014 (18)	Italy	Cross-sectional	9

### 3.2 Characteristics of participants

The characteristics of the included studies are shown in Table 1. 12 studies included a total of 1,460 patients with COPD. The sample size ranged from 34 (13) to 309 (15). The mean age of the participants was 51.74–71.92 years. Except for one study (4), which did not indicate the gender ratio, all the other 11 studies had a clear gender ratio, with a male to female ratio of 79:21 (1,102 to 296). The prevalence of thyroid dysfunction was demonstrated in 12 studies, 6 of which compared the clinical characteristics of COPD with and without thyroid dysfunction, respectively (4, 6–8, 12, 14, 15). Clinical characteristics included the following features: age, sex ratio, body mass index (BMI), smoking index, FEV1%, FVC%, PCO2, PO2, albumin. Five of these studies (8, 11, 12, 14, 15) specifically presented the prevalence of non-thyroidal disease syndrome (NTIS) in COPD. Two studies have examined the difference in the prevalence of thyroid dysfunction in COPD patients during two different periods: stable and acute exacerbation (11, 16). Three studies (4, 7, 11) included a control group of 90 participants without COPD.

### 3.3 Prevalence of thyroid dysfunction among COPD patients

**Figure 2:**
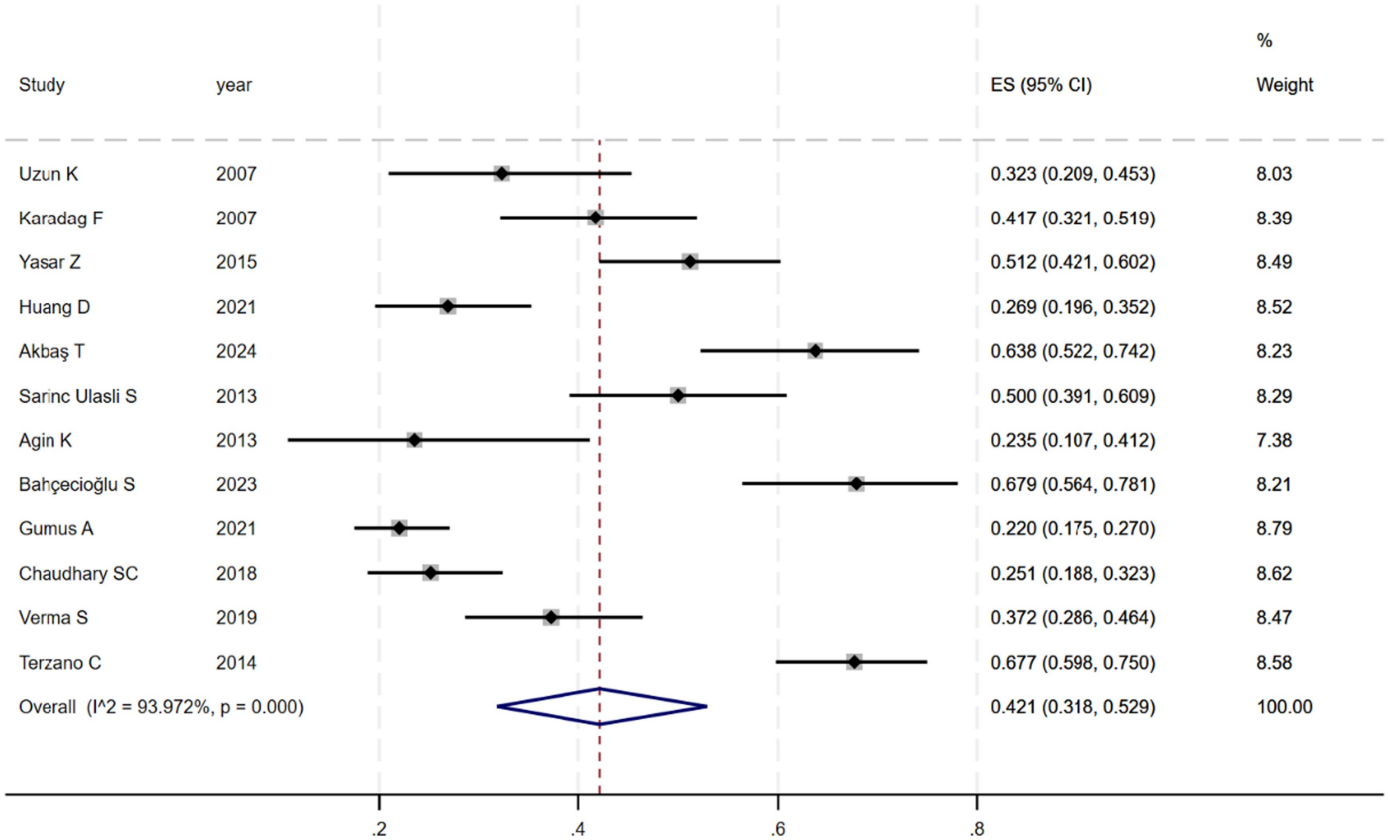
Prevalence of thyroid dysfunction among COPD patients.

**Figure 3:**
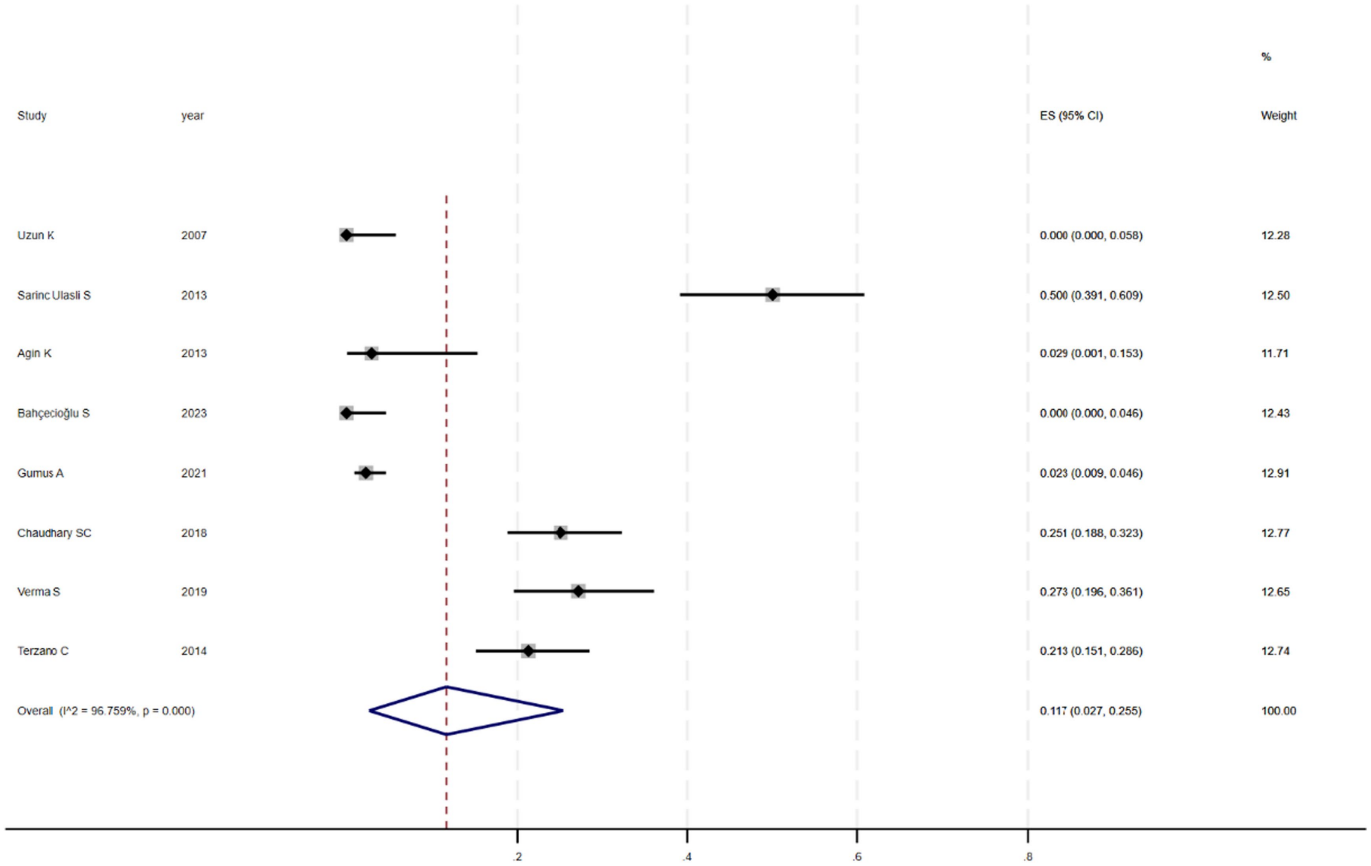
Prevalence of hypothyroidism in COPD.

**Figure 4:**
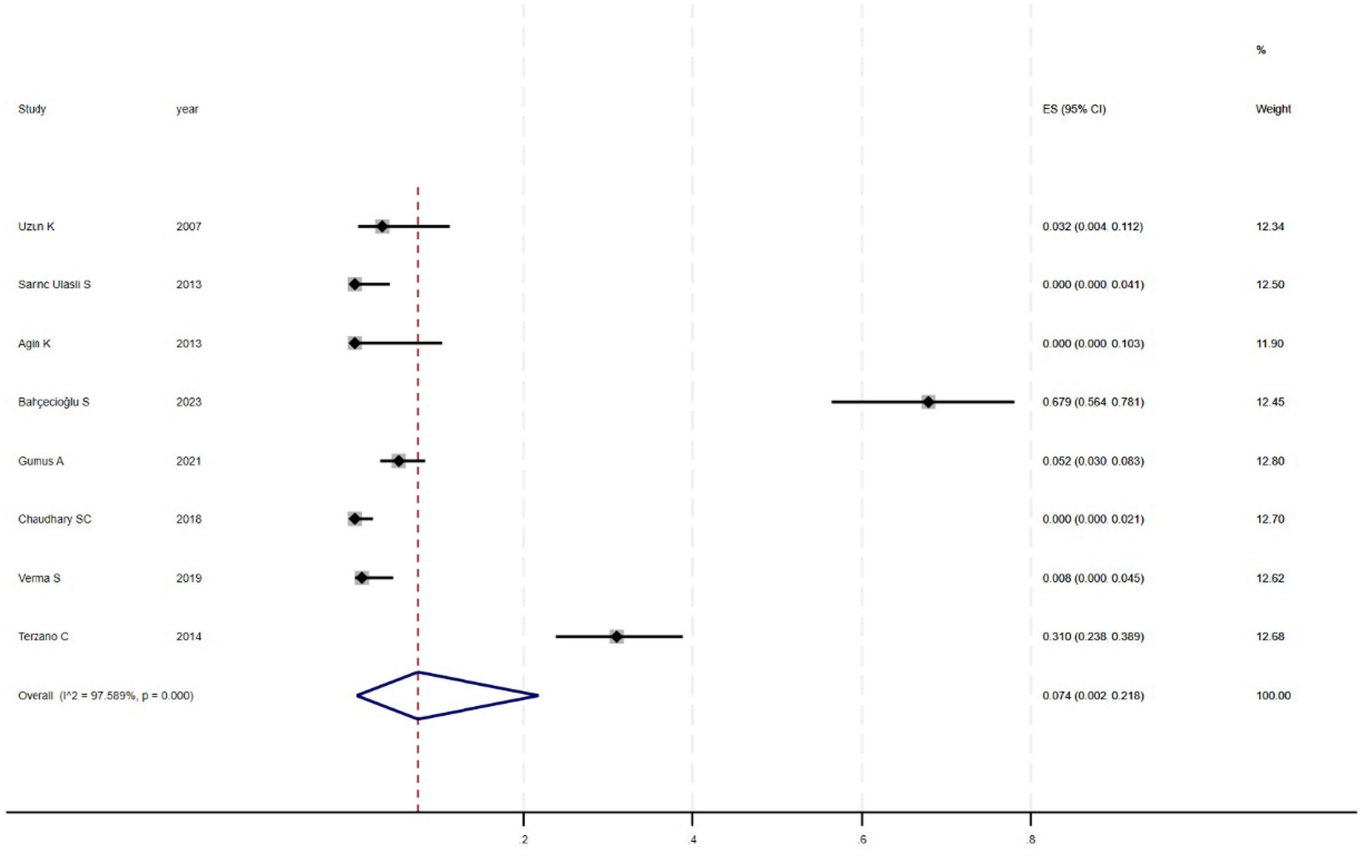
Prevalence of hyperthyroidism in COPD.

**Figure 5:**
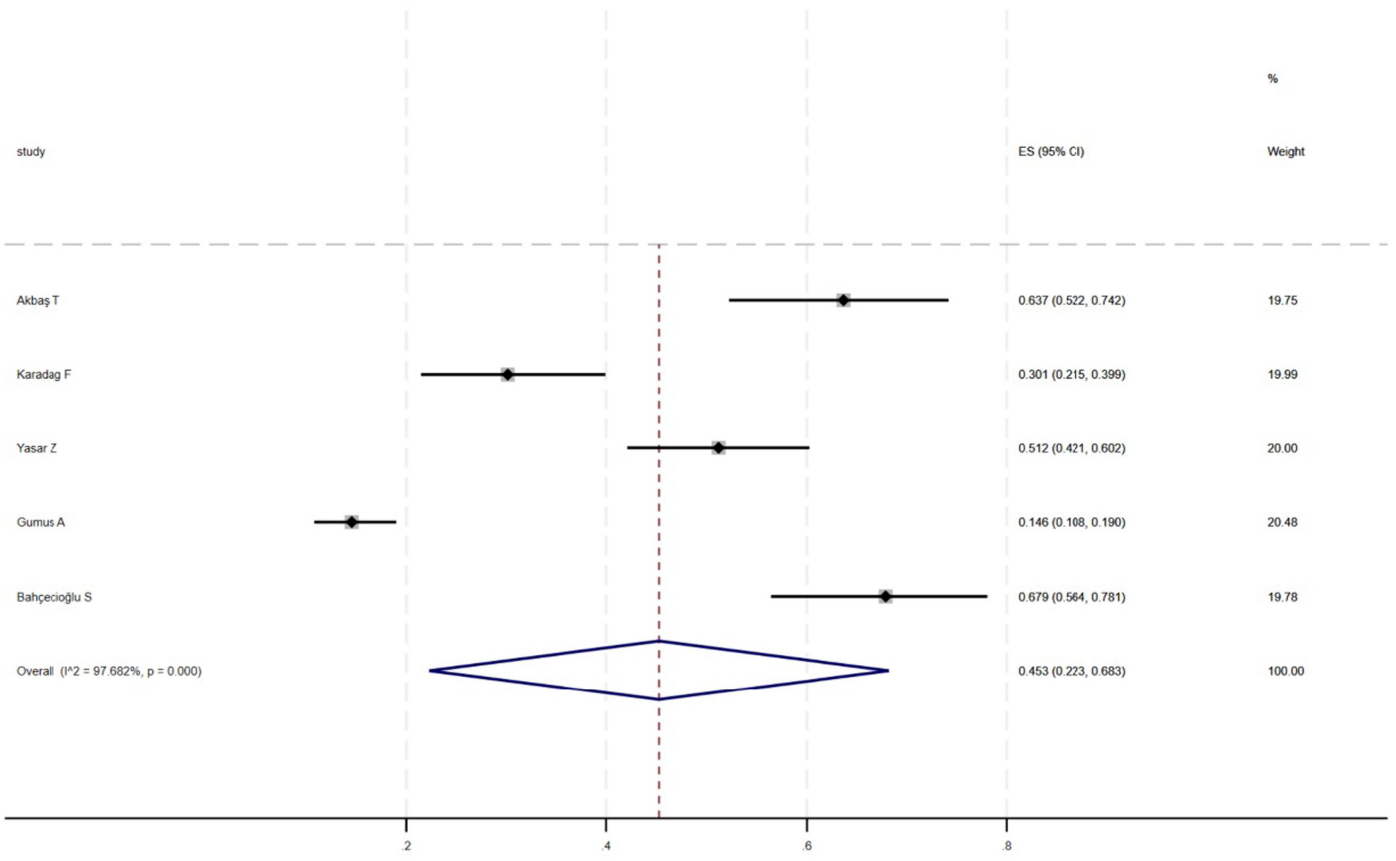
Prevalence of NTIS in COPD.

**Figure 6:**
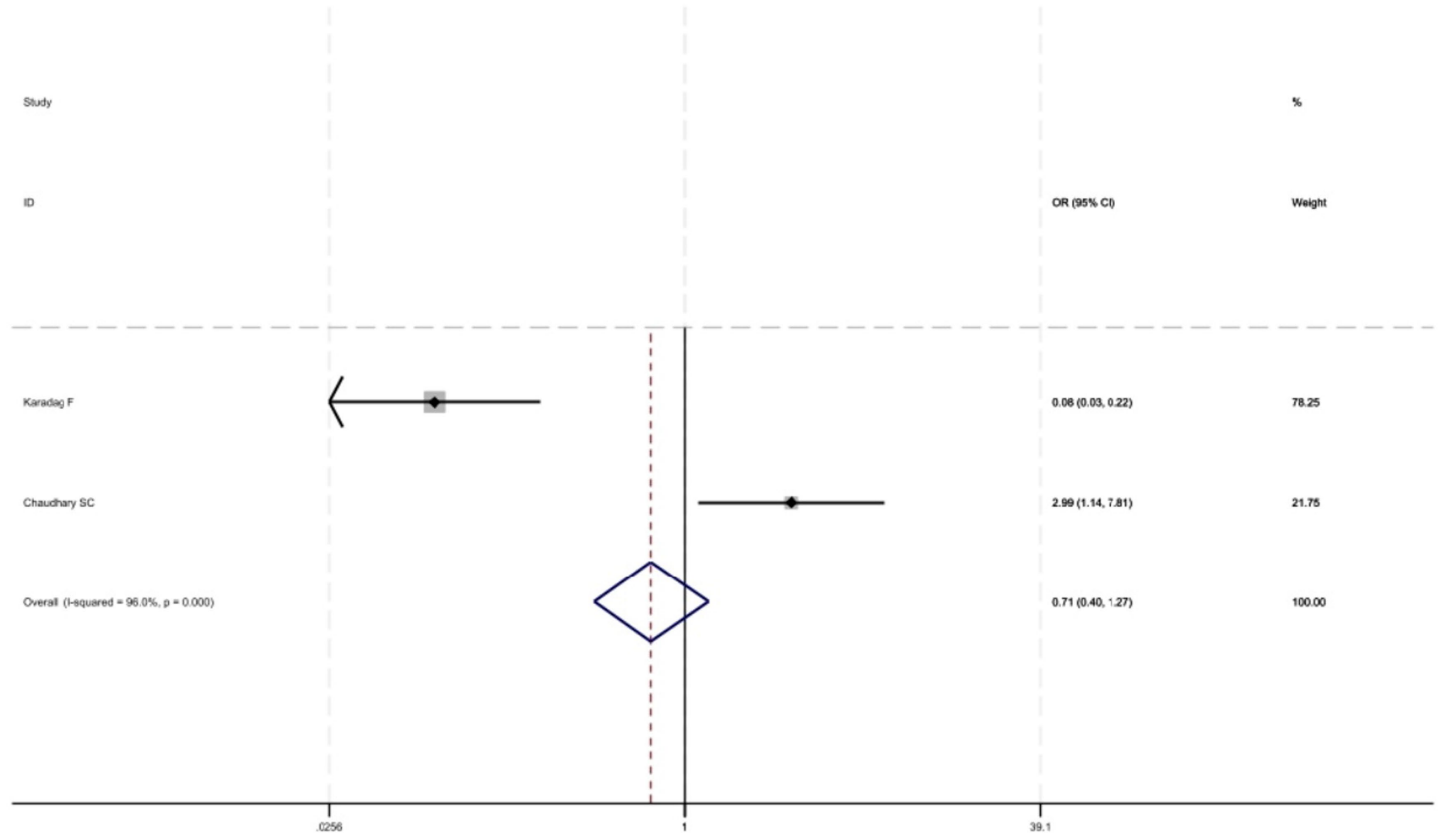
Comparison of prevalence of thyroid dysfunction in stable and acute exacerbations of COPD.

Figure 2 shows that among the 12 eligible studies included in the analysis, the overall prevalence of thyroid dysfunction among COPD patients was 42.1% (95% CI: 31.8–52.9). Because there was significant heterogeneity among the included studies (*I*^2^ = 93.972%; *p* < 0.01), meta-regression analysis was performed to explore the source of heterogeneity. Meta-regression analyses were performed for sample size, publication year, age, geographic distribution, and gender ratio. Table 3 presents the results of the meta-regression analysis, which indicated that the age of COPD patients (*p* = 0.035) and the geographic distribution of COPD patients (South and East Asia) (*p* = 0.047) were significantly associated with the heterogeneity of the included studies. Eight studies (4, 7, 13–18) (*n* = 1,018) specifically reported the number of patients with hyperthyroidism and hypothyroidism in COPD, with Figure 3 showed that the overall prevalence of hypothyroidism was 11.7% (95% CI: 2.7–25.5), and Figure 4 indicated that the overall prevalence of hyperthyroidism was 7.4% (95% CI: 2–21.8). Additionally, five studies (8, 11, 12, 14, 15) (*n* = 617) examined the prevalence of NTIS in COPD, with Figure 5 showing that the prevalence of NTIS in COPD patients was 45.3% (95% CI: 22.3–68.3). We also compared the prevalence of thyroid dysfunction in COPD patients during the stable and acute exacerbation periods and found no difference in prevalence between the two periods (OR = 0.710, 95% CI, 0.396–1.272, *p* = 0.2249) (Figure 6).

**Table 3:** Results of meta-regression analysis.

Variable	Coefficient	SE	*P* value
Sample size	−0.0009	0.0006	0.193
Year	0.0026	0.01	0.796
Age (y)	0.024	0.008	0.035
Sex (% male)	−0.0112	0.0075	0.171
Region			
Europe (*n* = 1)	Reference		
Middle East (*n* = 8)	0.789	0.121	0.156
South and East Asia (*n* = 3)	0.682	0.113	0.047

Europe: Italy; Middle East: Turkey and Iran; South and East Asia: India and China.

### 3.4 Comparison of clinical features in COPD patients with and without thyroid dysfunction

Six studies (6–8, 12, 14, 15) were conducted to compare and analyse the relevant clinical characteristics and indicators of patients with and without thyroid dysfunction in COPD (Table 4). As shown in the table, there was no statistically significant difference between the two groups in general characteristics such as age, BMI, sex ratio (proportion of males), and smoking index (*p* > 0.05). There was a statistically significant difference in Pulmonary function test (PFT) in terms of Forced Expiratory Volume in One Second as a percentage of predicted value (FEV1%) (*p* = 0.027), with higher FEV1% values in patients with thyroid dysfunction in COPD, whereas forced vital capacity as a percentage of predicted value (FVC%) did not differ between the two groups. Comparison of blood gas analysis parameters between the two groups revealed that partial pressure of carbon dioxide (PCO2) was lower in the group with thyroid dysfunction (*p* = 0.048), while PO2 was not statistically different between the two groups. Finally, we observed a difference in albumin levels, with the normal thyroid function group having higher albumin values (*P* = 0.00).

**Table 4:** Comparison of clinical characteristics between COPD patients without and with thyroid dysfunction.

Variable	MD (95% CI)	*p*-value**	I-square
Age (y)	−0.029 (−0.198, 0.139)	0.732	66%
BMI (kg/m2)	−0.182 (−0.387, 0.023)	0.081	0.00%
Sex (% male)*	0.930 (0.609, 1.420)	0.736	13.50%
Cigarette (pack/year)	0.217 (−0.011, 0.445)	0.062	83.30%
FEV1 (%)	−0.256 (−0.483, −0.029)	0.027	0.00%
FVC (%)	−0.022 (−0.249, 0.206)	0.853	84.30%
PO2 (mmHg)	0.102 (−0.134, 0.038)	0.397	87.00%
PCO2 (mmHg)	0.212 (0.002, 0.422)	0.048	76.10%
Albumin (g/L)	0.832 (0.522, 1.141)	0.000	91.90%

*Expressed as odds ratio (OR) (95% CI). **p-value for the comparison of the two groups of indicators.

### 3.5 Publication bias and sensitivity analysis

**Figure 7:**
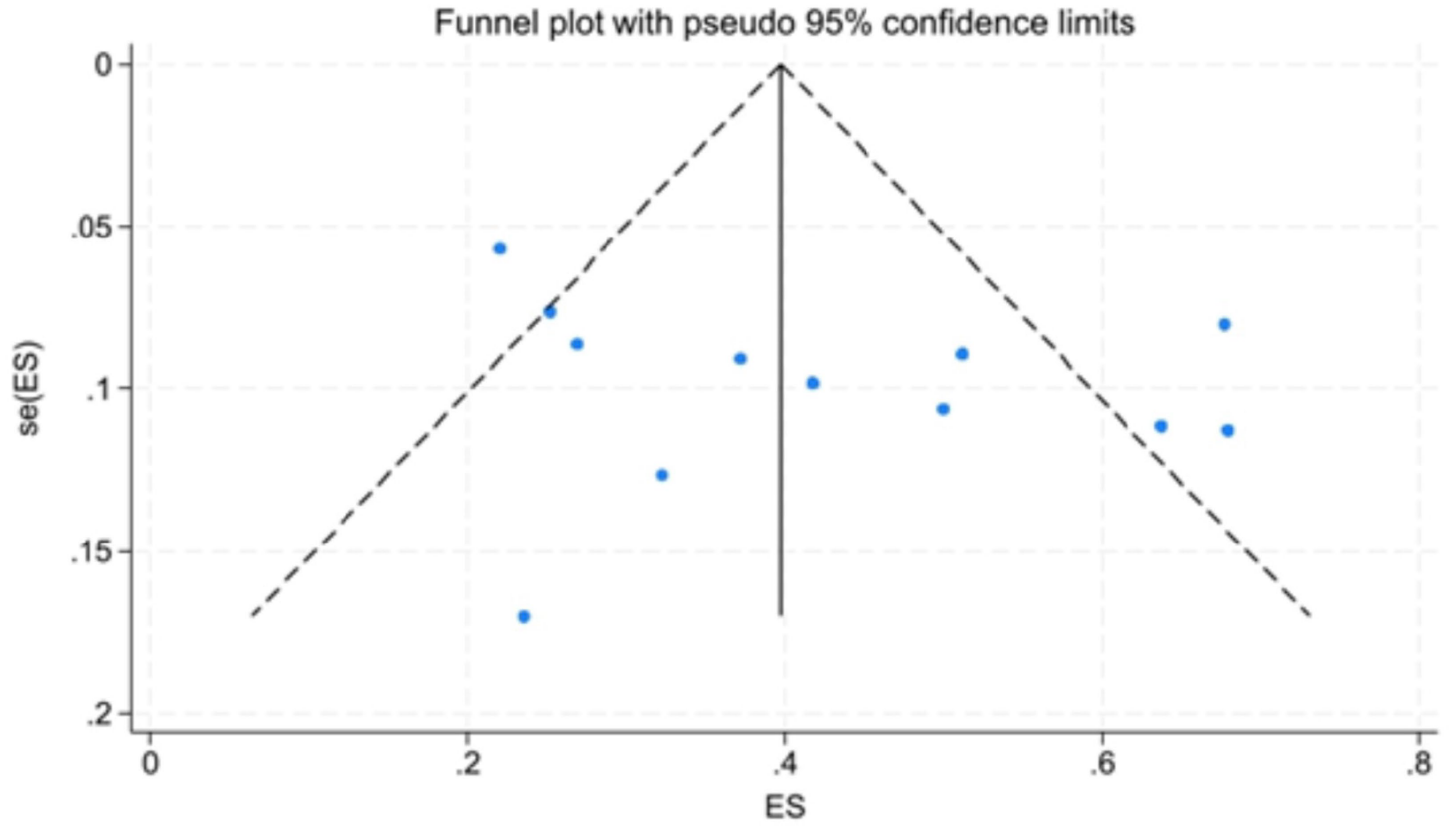
Funnel plot.

**Figure 8:**
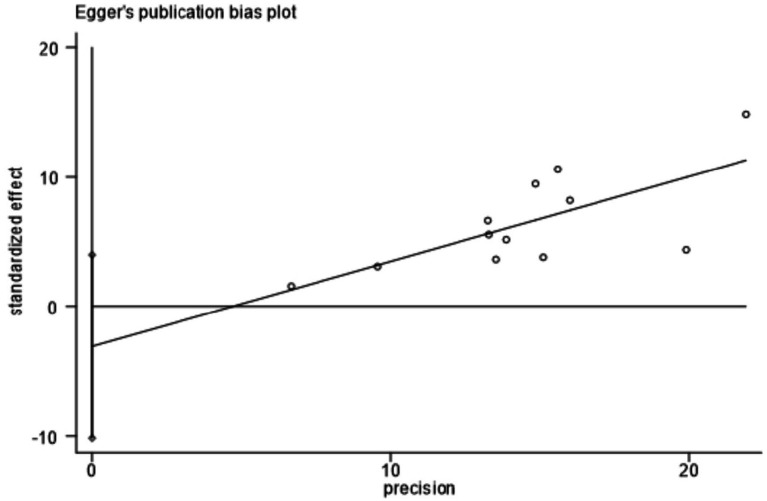
Egger test.

**Figure 9:**
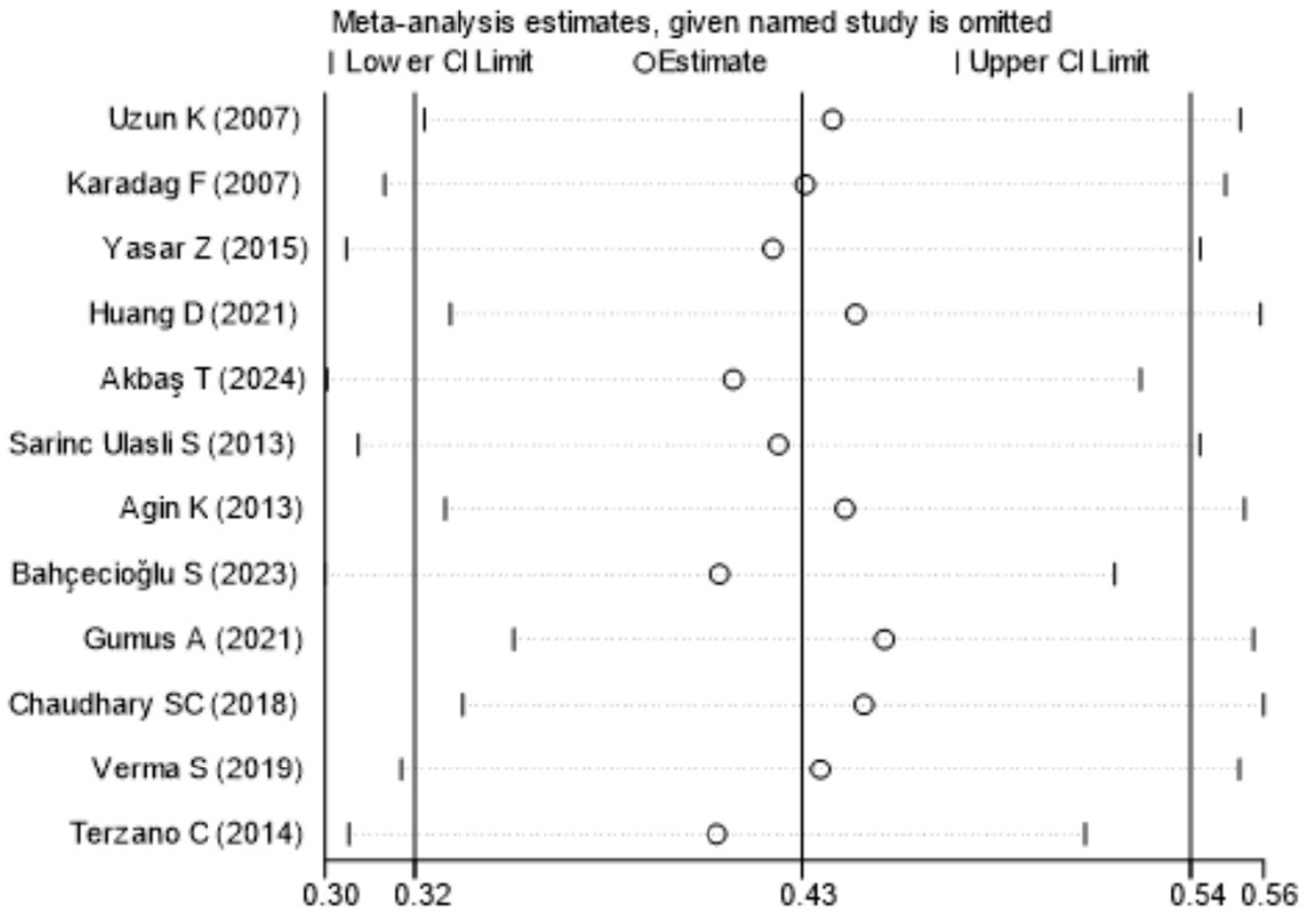
Sensitivity analysis.

Figure 7 showed that the visual inspection of the funnel plot was largely symmetrical. The results of the Egger’s test in Figure 8 indicated that publication bias had no significant impact (*P* value = 0.354 > 0.05). The results of the sensitivity analysis in Figure 9 demonstrated that no single study fundamentally altered the overall prevalence of all outcomes.

## 4 Discussion

Chronic obstructive pulmonary disease (COPD) is a common respiratory chronic disease with high global morbidity and mortality. Systemic inflammation originating from the lungs can lead to the development of COPD comorbidities, both in the stabilised and acute exacerbation phases (19), and this associated comorbidity is a key prognostic factor influencing the outcome of COPD (1). Recently, several studies have shown that thyroid dysfunction are more common in patients with COPD. A large population-based study conducted by García-Olmos et al. in the city of Madrid, Spain (198,670 patients) showed a prevalence of COPD of 3.2%, whereas the prevalence of thyroid dysfunction in patients with COPD was 14.21% (20). This meta-analysis combining 12 studies found that the overall prevalence of thyroid dysfunction among COPD patients was 42.1%. It is close to the prevalence of 45% in the meta-study of Arrey Agbor DB. This meta-analysis conducted a meta-regression analysis to explore the sources of heterogeneity due to significant heterogeneity among the included studies. Table 3 showed that the age of COPD patients and the geographic region (South and East Asia) were the two factors that contributed most to the heterogeneity of the results. This indicated that there were significant differences in thyroid function among COPD patients of different ages in different regions. In the future, when designing prospective studies, it should be ensured that patients from various age groups and regions are included to avoid biases. Thyroid dysfunction in COPD patients could present as subclinical hypothyroidism, hypothyroidism, hyperthyroidism, and non-thyroidal illness syndrome. This meta-analysis showed that the overall prevalence of hypothyroidism in COPD was 11.7%, the overall prevalence of hyperthyroidism was 7.4%, and the prevalence of NTIS was 45.3%. This indicated that the prevalence of thyroid dysfunction among COPD patients was relatively high, with NTIS being the most common thyroid disorder. Bacakoğlu et al. reported the prevalence of NTIS in COPD patients to be 68% (21), which is higher than the 45.3% found in this meta-analysis. This shows that the prevalence of thyroid dysfunction is high in COPD and NTIS is the most common thyroid dysfunction. Non-thyroidal illness syndrome (NTIS) can be defined as abnormal levels of thyroid hormones in response to starvation, stress, or severe illness, and low T3 is the mildest and most common form of NTIS and is seen in about 35–70% of hospitalised patients (22, 23). NTIS consists of a normal or decreased total thyroxine and free thyroxine (TT4 and FT4, respectively), with normal or decreased total thyroxine (TT3) and free thyroxine (FT3) are reduced, and thyroid stimulating hormone (TSH) levels are usually normal (24). Therefore, in clinical practice, thyroid function in COPD patients often showed a decrease in only the T3 level.

The relationship between COPD and thyroid function has recently received increasing attention. The mechanisms by which thyroid dysfunction occurs in chronic obstructive pulmonary disease have not been fully investigated, but may involve hypoxaemia, systemic inflammation and glucocorticoid use (25). The thyroid and lungs, both from Nkx2.1 cells in the same endodermal layer, may explain the histo-anatomical basis for their mutual influence (25). The mechanisms underlying thyroid dysfunction in COPD are not yet fully understood but may involve hypoxemia, systemic inflammatory factors, glucocorticoid use, smoking, and infections (26). The possible mechanisms of thyroid dysfunction in COPD are as follows: 1. Hypoxemia: Hypoxemia can cause hypothalamic–pituitary dysfunction, leading to a delayed response of thyroid-stimulating hormone (TSH) to thyrotropin-releasing hormone (TRH), which affects the synthesis and metabolism of peripheral thyroid hormones (27). Additionally, hypoxemia reduces the activity of peripheral 5′-deiodinase, decreasing the conversion of T4 to the bioactive hormone T3 (24). It can also increase tissue utilization of T3, lowering its levels in the blood (28), and decrease the levels of thyroxine-binding globulin, which affects thyroid hormone binding and reduces T3 levels (18). 2. Systemic Inflammatory Factors: Patients with COPD often exhibit elevated levels of systemic inflammatory markers such as interleukin-6 (IL-6), interleukin-1 (IL-1), and tumor necrosis factor-alpha (TNF-*α*) (29). These inflammatory factors can inhibit the secretion of TSH and T3, as well as the synthesis of thyroid hormone-binding proteins. They may also reduce the expression of liver enzyme iodothyronine deiodinase type 1 mRNA, an enzyme that converts T4 to T3 (30), thus lowering T3 levels. Various factors such as hypoxemia, smoking, and infections can stimulate the release of systemic inflammatory factors. Therefore, some studies consider the pathway of systemic inflammatory factors to be one of the main mechanisms for the occurrence of thyroid dysfunction in COPD (27). 3. Glucocorticoids: Glucocorticoids are often used during the course of COPD. High levels of glucocorticoids can inhibit the pituitary’s response to TRH, leading to central hypothyroidism and affecting thyroid function. They can also regulate peripheral hormone metabolism by shifting the deiodination of T4 from activation to inactivation, and they can redistribute T4 and T3 in the blood and tissue spaces (31, 32). 4. Smoking: Smoking is a significant risk factor for COPD. The large number of free radicals in tobacco smoke can damage airway structures and promote inflammation (33). Reports indicate that smoking can have both inhibitory and stimulatory effects on thyroid function, with an ability to raise T3 levels (34). 5. Infections: COPD patients often experience respiratory infections that lead to acute exacerbations. When infection occurs, organismal stress promotes activation of the neuroendocrine system, increasing the synthesis and secretion of catecholamines, glucocorticoids, and cortisol, and inhibiting the release of TSH. Infections can similarly decrease T3 levels by inhibiting the conversion of T4 to T3 and affecting thyroid hormone binding (28). Severe infections can stimulate the release of large amounts of inflammatory cytokines, impacting the synthesis, secretion, and metabolism of thyroid hormones. Repeated infections and chronic depletion result in decreased plasma albumin levels and reduced synthesis of thyroid-binding globulin, affecting the binding of thyroid hormones and lowering T3 levels (35). In summary, multiple factors influence thyroid function in COPD, and the effects are not singular. For instance, hypoxemia, infections, and smoking can all trigger the release of inflammatory factors. Moreover, both hypoxemia and infections can induce a stress state, increasing the synthesis and secretion of glucocorticoids, leading to thyroid dysfunction. These mechanisms help explain the high prevalence of thyroid dysfunction, particularly NTIS, among COPD patients. Thyroid dysfunction also affects COPD. Brüssel T et al. showed that thyroid dysfunction can affect COPD through upper airway obstruction, decreased respiratory muscle tone, central and obstructive sleep apnoea, alveolar hypoventilation and pleural effusion (36).

This meta-analysis also compared the prevalence of thyroid dysfunction in COPD patients in acute exacerbation and stable phase was not different (*p* > 0.05). This may be because COPD is a chronic systemic inflammatory disease with long-term hypoxemia, leading to the adaptation and regulatory feedback of the hypothalamic–pituitary-thyroid axis. Whereas Karadag et al. study found that in the stable phase of COPD is the prevalence of thyroid dysfunction was 20% and in acute exacerbation the prevalence was as high as 70% (11). Gupta Madhuri et al. found that NTIS was more pronounced in patients with AECOPD as compared to patients with stable disease (37). In this meta-analysis, only 2 studies (11, 16) were included in the analysis, which is an insufficient sample size, and more data with studies are needed in the future to clarify whether there is a difference in prevalence between the two periods.

The severity of airway obstruction in COPD is associated with thyroid dysfunction (4), i.e., thyroid function can also affect pulmonary function, consistent with the study by Gao et al. (38). Dimopoulou et al. found a positive correlation between TT3, TT4, TT3%, and TT4 and FEV1 when FEV1 was less than 50% (24). Dan Huang et al. analysed FVC, FEV1 and FEV1%pred in AECOPD patients with normal thyroid hormone levels than in AECOPD patients with abnormal thyroid hormone levels. In this paper, from the inclusion of 2 studies, it was found that FEV1% was higher in patients with thyroid dysfunction in COPD than in patients in the normal thyroid group, and FVC did not differ between the two groups, which is inconsistent with the findings of the above studies. Because the FEV1% data in the Dan Huang et al. study were non-normal, it is not included in this meta-analysis when examining the FEV1%. It has also been shown that there is no significant correlation between hormone levels and pulmonary function in COPD patients (39). Future large-scale cross-sectional surveys of data or prospective studies are needed to clarify the correlation between lung function and thyroid hormones. Neither Banks et al. nor Gow et al. found any correlation between thyroid hormone levels and arterial blood gas measurements in patients with COPD (27, 40). The present meta-study found that PCO2 was lower in the thyroid dysfunction group in COPD compared to the normal thyroid dysfunction group, and that lowering the partial pressure of carbon dioxide may act as a compensation for the hypoxia in the body. The lower albumin index in the thyroid dysfunction group in COPD suggests that the thyroid dysfunction group may be more susceptible to malnutrition, and muscle protein catabolism, which can lead to muscle atrophy and reduced muscle strength, affecting activity tolerance and aggravating the condition of COPD.

Thyroid dysfunction may affect the prognosis of will COPD. Normal physiological activity of thyroid hormones is largely realized by T3. A decrease in serum T3 levels can lead to multi-organ dysfunction (41). In patients with thyroid dysfunction, a mild reduction in T3 levels serves as a protective adaptation that reduces energy expenditure and alleviates symptoms. However, a significant drop in T3 levels indicates severe pathological damage and suggests the disease may progress towards a more severe phase (42). Ulasli et al. found that TSH is a determinant of the frequency of AECOPD (7). Yasar et al. found is that NTIS may be an independent predictor of prolonged deconditioning in endotracheal intubated COPD patients (12). Correction of hypothyroidism has been reported to help patients withdraw from the ventilator (43). Patients with NTIS have a level that predicts ICU prognosis in COPD patients. The degree of thyroid hormone alteration is related to the severity of the disease, and thyroid hormone levels are significantly lower in critically ill COPD patients with higher Acute Physiological and Chronic Health Evaluation (APACHE) II scores (44). The above studies suggest that thyroid dysfunction may affect the prognosis of COPD patients by affecting their lung function, increasing the frequency of acute exacerbations, and prolonging the time to withdraw and extubate intubated patients. Therefore, in clinical work, thyroid hormone levels should be monitored in COPD patients, which can help identify critically ill patients, formulate appropriate withdrawal and extubation strategies, strengthen healthcare management, and improve prognosis.

Due to the high prevalence of thyroid dysfunction among COPD patients and its impact on prognosis, clinical attention should be prioritized. Thyroid function should be screened promptly in the following situations:1. COPD patients experiencing unexplained weight changes (such as weight gain or loss), fatigue, drowsiness, emotional changes (like depression or anxiety), sweating or sensitivity to cold, palpitations, or hand tremors; 2. Patients with comorbid autoimmune diseases, such as rheumatoid arthritis or systemic lupus erythematosus; 3. Those with more severe disease or frequent acute exacerbations; 4. Patients using glucocorticoids long-term or those aged 65 and older; 5. Patients with a history of smoking or have infections; 6 patients with a family history of thyroid disease.

There is a controversy over the need to intervene in the treatment of thyroid hormone level abnormalities in patients with COPD. Some studies have suggested that thyroid dysfunction in COPD is a compensatory way of adapting the body to the disease to counteract excessive catabolism and proteolysis, and that when the condition improves, thyroid function may return to normal (11). However, it has also been suggested that thyroid dysfunction should be corrected as soon as possible may improve the condition and prognosis of COPD patients (45). However, there is a lack of sufficiently strong evidence to support both of the above scenarios, and future large-scale studies are needed for clarification.

Therefore, in the future, we can focus on cohort studies examining thyroid function changes in COPD patients with thyroid dysfunction, and conduct randomized controlled trials (RCTs) to assess whether early intervention for thyroid dysfunction in COPD patients can benefit the frequency of acute exacerbations.

In summary, this meta-analysis found a high prevalence of thyroid dysfunction in COPD, predominantly of the NTIS type, and that thyroid dysfunction may affect pulmonary function, with the risk of protein loss and malnutrition. Early dynamic monitoring of thyroid hormone levels in patients with COPD, especially in patients with respiratory failure requiring endotracheal intubation and mechanical ventilation in COPD, and multidisciplinary collaborative management with endocrinology, if necessary, will improve the patient’s condition and prognosis.

### 4.1 Limitations

This meta-analysis has several limitations. First, it included 12 studies with a total of 1,460 patients, which is a limited sample size. Second, seven of the included studies were conducted in Turkey; while not heterogeneous, regional influences may affect the outcome measures. Additionally, the sample sizes for studies comparing the prevalence of thyroid dysfunction in different phases of COPD and the clinical characteristics of patients with and without thyroid dysfunction were small, and the assessment criteria were not consistently evaluated. This meta-analysis included seven cross-sectional studies, thus causal relationships between COPD and thyroid function could not be clearly established. Studies with negative results were not included in this systematic review. Moreover, the inconsistent conclusions regarding the relationship between thyroid dysfunction and lung function pose difficulties in interpreting clinical significance and are not conducive to guiding clinical strategies. Finally, there is controversy over whether interventions for thyroid dysfunction are related to the prognosis of COPD, making it uncertain whether treating thyroid dysfunction benefits COPD patients. Given these limitations, there is a need for large prospective studies to clarify the relationship between COPD and thyroid dysfunction and the impact of thyroid dysfunction on the prognosis of COPD patients.

## Data Availability

The original contributions presented in the study are included in the article/Supplementary material, further inquiries can be directed to the corresponding author.
