# Supplementary material for: Prevalence and clinical characterisation of thyroid dysfunction in COPD: a systematic review and meta-analysis
Source: Front Med (Lausanne). 2025 Apr 30;12:1571165. doi: 10.3389/fmed.2025.1571165 (PMC12082834; doi:10.3389/fmed.2025.1571165)

Appendix 1. The search strategy for articles on thyroid dysfunction prevalence in COPD patients

PubMed

| # | Searches | Results |
| --- | --- | --- |
| #1 | ("Pulmonary Disease, Chronic Obstructive"[Mesh]) OR (((((((((((Pulmonary Disease, Chronic Obstructive) OR (Chronic Obstructive Pulmonary Diseases)) OR (COAD)) OR (COPD)) OR (Chronic Obstructive Airway Disease)) OR (Chronic Obstructive Pulmonary Disease)) OR (Airflow Obstruction, Chronic)) OR (Chronic Obstructive Lung Disease)) OR (Airflow Obstructions, Chronic)) OR (Chronic Airflow Obstructions)) OR (Chronic Airflow Obstruction)) |  |
| #2 | ("Thyroid Hormones"[Mesh]) OR ((((Thyroid Hormones) OR (Thyroid Hormone)) OR (Hormones, Thyroid)) OR (Hormone, Thyroid)) |  |
| #3 | #1 and #2 | 187 |

Web of Science

| # | Searches | Results |
| --- | --- | --- |
| #1 | Pulmonary Disease, Chronic Obstructive (Topic) OR Chronic Obstructive Pulmonary Diseases (Topic) OR COAD (Topic) OR COPD (Topic) OR Chronic Obstructive Airway Disease (Topic) OR Chronic Obstructive Pulmonary Disease (Topic) OR Airflow Obstruction, Chronic (Topic) OR Chronic Obstructive Lung Disease (Topic) OR Airflow Obstructions, Chronic (Topic) OR Chronic Airflow Obstructions (Topic) OR Chronic Airflow Obstruction (Topic) and Preprint Citation Index (Exclude – Database) |  |
| #2 | Thyroid Hormones (Topic) OR Thyroid Hormone (Topic) OR Hormones, Thyroid (Topic) OR Hormone, Thyroid (Topic) and Preprint Citation Index (Exclude – Database) |  |
| #3 | #1 and #2 | 252 |

EMBASE

| # | Searches | Results |
| --- | --- | --- |
| #1 | 'pulmonary disease, chronic obstructive'/exp OR 'pulmonary disease, chronic obstructive' OR (pulmonary AND ('disease,'/exp OR disease,) AND chronic AND obstructive) OR (chronic AND obstructive AND pulmonary AND diseases) OR coad OR copd OR (chronic AND obstructive AND airway AND disease) OR (chronic AND obstructive AND pulmonary AND disease) OR (airflow AND obstruction, AND chronic) OR (chronic AND obstructive AND lung AND disease) OR (airflow AND obstructions, AND chronic) OR (chronic AND airflow AND obstructions) OR (chronic AND airflow AND obstruction) |  |
| #2 | 'thyroid hormones'/exp OR 'thyroid hormones' OR (thyroid AND ('hormones'/exp OR hormones)) OR (thyroid AND hormone) OR (hormones, AND thyroid) OR (hormone, AND thyroid) |  |
| #3 | #1 and #2 | 293 |

Cochrane Library

| # | Searches | Results |
| --- | --- | --- |
| #1 | MeSH descriptor: [Pulmonary Disease, Chronic Obstructive] explode all trees |  |
| #2 | (Pulmonary Disease, Chronic Obstructive) OR (Chronic Obstructive Pulmonary Diseases) OR (COAD) OR (COPD) OR (Chronic Obstructive Airway Disease) |  |
| #3  #4  #5  #6  #7  #8  #9 | (Chronic Obstructive Pulmonary Disease) OR (Airflow Obstruction, Chronic) OR (Chronic Obstructive Lung Disease) OR (Airflow Obstructions, Chronic):kw OR (Chronic Airflow Obstructions)  (Chronic Airflow Obstruction)  #1 or #2 or #3 or #4  MeSH descriptor: [Thyroid Hormones] explode all trees  (Thyroid Hormones) OR (Thyroid Hormone) OR (Hormones, Thyroid) OR (Hormone, Thyroid)  #6 or #7  #5 and #8 | 43 |

Appendix 2

**Cross-Sectional/Prevalence Study Quality**

| **Item** | **Yes** | **No** | **Unclear** |
| --- | --- | --- | --- |
| 1) Define the source of information (survey, record review) |  |  |  |
|  | | | |
| 2) List inclusion and exclusion criteria for exposed and unexposed subjects (cases and controls) or refer to previous publications |  |  |  |
|  | | | |
| 3) Indicate time period used for identifying patients |  |  |  |
|  | | | |
| 4) Indicate whether or not subjects were consecutive if not population-based |  |  |  |
|  | | | |
| 5) Indicate if evaluators of subjective components of study were masked to other aspects of the status of the participants |  |  |  |
|  | | | |
| 6) Describe any assessments undertaken for quality assurance purposes (e.g., test/retest of primary outcome measurements) |  |  |  |
|  | | | |
| 7) Explain any patient exclusions from analysis |  |  |  |
|  | | | |
| 8) Describe how confounding was assessed and/or controlled. |  |  |  |
|  | | | |
| 9) If applicable, explain how missing data were handled in the analysis |  |  |  |
|  | | | |
| 10) Summarize patient response rates and completeness of data collection |  |  |  |
|  | | | |
| 11) Clarify what follow-up, if any, was expected and the percentage of patients for which incomplete data or follow-up was obtained |  |  |  |

Appendix 3


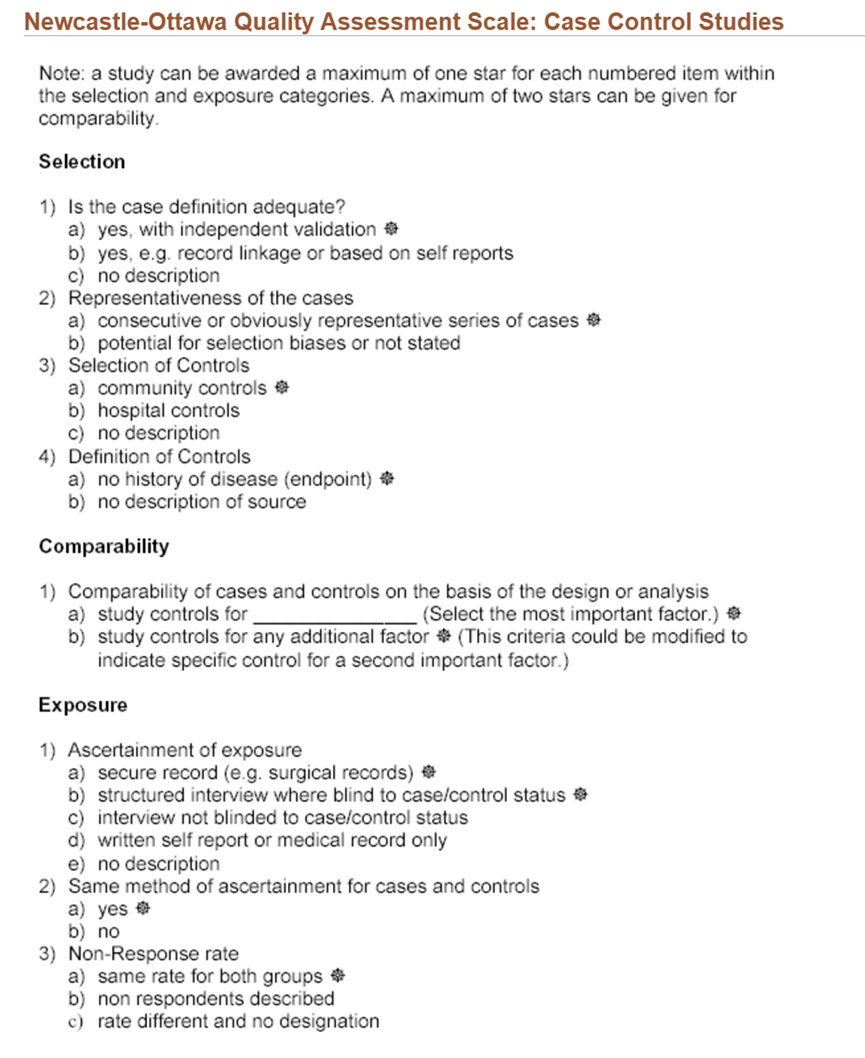


Appendix 4


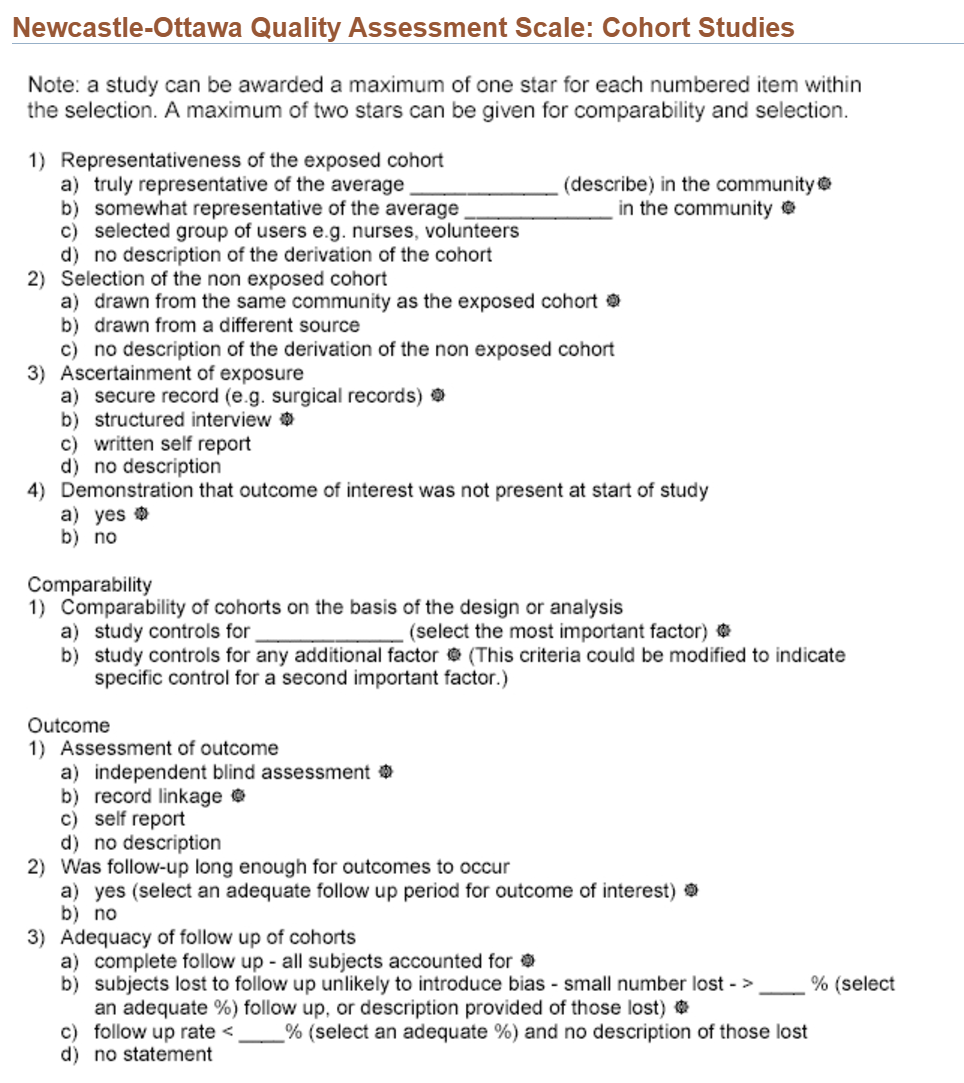

Supplement: Supplementary file 2 [file Table_2.docx]
